# 12-(4-Methoxy­phen­yl)-10-phenyl-3,4,5,6,8,10-hexa­azatricyclo­[7.3.0.0^2,6^]dodeca-1(9),2,4,7,11-penta­ene

**DOI:** 10.1107/S160053681000869X

**Published:** 2010-03-13

**Authors:** Mukesh M. Jotani, Rina D. Shah, Edward R. T. Tiekink

**Affiliations:** a Department of Physics, Bhavan’s Sheth R. A. College of Science, Ahmedabad, Gujarat 380 001, India; b Department of Chemistry, M. G. Science Institute, Navrangpura, Ahmedabad, Gujarat 380 009, India; c Department of Chemistry, University of Malaya, 50603 Kuala Lumpur, Malaysia

## Abstract

In the title compound, C_19_H_14_N_6_O, the fused 12-membered tetra­zolo/pyrimidine/pyrrole ring system is almost planar (r.m.s. deviation = 0.013 Å). The 4-methoxy­phenyl and phenyl substituents on the pyrrole ring are both twisted with respect to the fused-ring system [dihedral angles = 25.39 (18) and 36.42 (18)°, respectively]. Intra­molecular C—H⋯N inter­actions occur. In the crystal, mol­ecules pack into layers in the *ac* plane and these are connected along the *b* axis *via* C—H⋯π and π–π [centroid–centroid separation = 3.608 (3) Å] inter­actions.

## Related literature

For background to the biological activity of fused tetra­zolopyrimidines, see: Shishoo & Jain (1992 ▶); Desai & Shah (2006 ▶). For related structures, see: Jotani *et al.* (2010*a* ▶,*b* ▶); Shah *et al.* (2010 ▶). For semi-empirical quantum chemical calculations, see: Stewart (2009 ▶).
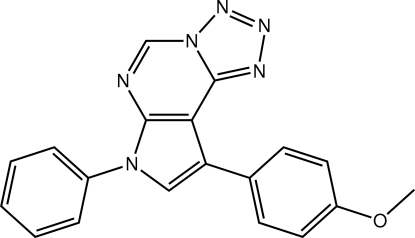


## Experimental

### 

#### Crystal data

C_19_H_14_N_6_O
*M* *_r_* = 342.36
Orthorhombic, 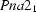

*a* = 9.3537 (7) Å
*b* = 23.6045 (19) Å
*c* = 7.1543 (6) Å
*V* = 1579.6 (2) Å^3^
*Z* = 4
Mo *K*α radiation
μ = 0.10 mm^−1^
*T* = 293 K
0.35 × 0.25 × 0.20 mm

#### Data collection

Bruker Kappa APEXII CCD diffractometer
Absorption correction: multi-scan (*SADABS*; Sheldrick, 1996 ▶) *T*_min_ = 0.967, *T*_max_ = 0.981
16155 measured reflections
1666 independent reflections
1344 reflections with *I* > 2σ(*I*)
*R*_int_ = 0.051

#### Refinement

*R*[*F* ^2^ > 2σ(*F* ^2^)] = 0.041
*wR*(*F* ^2^) = 0.142
*S* = 1.12
1666 reflections
236 parameters
1 restraint
H-atom parameters constrained
Δρ_max_ = 0.50 e Å^−3^
Δρ_min_ = −0.27 e Å^−3^

### 

Data collection: *APEX2* (Bruker, 2004 ▶); cell refinement: *SAINT* (Bruker, 2004 ▶); data reduction: *SAINT*; program(s) used to solve structure: *SHELXS97* (Sheldrick, 2008 ▶); program(s) used to refine structure: *SHELXL97* (Sheldrick, 2008 ▶); molecular graphics: *ORTEP-3* (Farrugia, 1997 ▶) and *DIAMOND* (Brandenburg, 2006 ▶); software used to prepare material for publication: *publCIF* (Westrip, 2010 ▶).

## Supplementary Material

Crystal structure: contains datablocks global, I. DOI: 10.1107/S160053681000869X/hb5355sup1.cif

Structure factors: contains datablocks I. DOI: 10.1107/S160053681000869X/hb5355Isup2.hkl

Additional supplementary materials:  crystallographic information; 3D view; checkCIF report

## Figures and Tables

**Table 1:** Hydrogen-bond geometry (Å, °) *Cg*1 is the centroid of the C14–C19 ring.

*D*—H⋯*A*	*D*—H	H⋯*A*	*D*⋯*A*	*D*—H⋯*A*
C8—H8⋯N4	0.93	2.50	3.257 (5)	138
C15—H15⋯N5	0.93	2.57	3.020 (5)	111
C11—H11⋯*Cg*1^i^	0.93	2.91	3.684 (5)	141
C13—H13c⋯*Cg*1^ii^	0.96	2.72	3.459 (5)	134

Symmetry codes: (i) 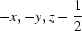; (ii) 
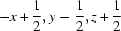
.

**Fig. 1.**
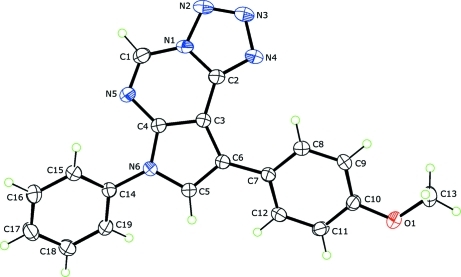
The molecular structure of (I) showing displacement ellipsoids at the 35% probability level.

**Fig. 2.**
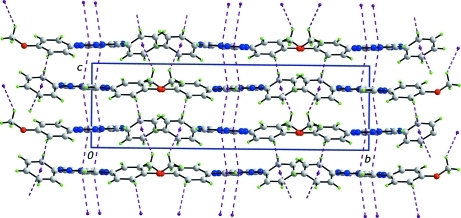
A view in projection down the a axis in (I), highlighting the C–H···π and π–π interactions (purple dashed lines). Colour code: O, red; N, blue; C, grey; and H, green.

## supplementary crystallographic information

### Comment

Interest in fused tetrazolopyrimidines relates, in part, to their biological activities (Shishoo & Jain, 1992; Desai & Shah, 2006). In continuation of complementary structural studies (Jotani *et al.* 2010*a*; Jotani *et al.* 2010*b*; Shah *et al.* 2010), the synthesis and X-ray crystal structure determination of the title compound, (I), are reported herein.

The molecule of (I) comprises a central pyrimidine ring (N1,N5,C1–C4) to which is fused a tetrazolo ring (N1–N4,C2) and a di-substituted pyrrole ring (N6,C3–C6), Fig. 1. These atoms form a plane with dihedral angles formed between the pyrimidine and the tetrazolo and pyrrole rings being 0.1 (3) and 1.5 (3) °, respectively; the dihedral angle formed between the tetrazolo and pyrrole rings is 1.6 (3) °. The r.m.s. deviation of the 12 non-hydrogen atoms comprising the fused ring system is 0.013 Å. The presence of intramolecular C–H···N interactions, Table 1, are noted and these result in the formation of S(6) and S(7) rings. The 4-methoxyphenyl and benzene substituents on the pyrrole ring are not co-planar with the fused-ring system as seen in the C3–C6–C7–C8 and C4–N6–C14–C15 torsion angles of 24.2 (9) and -39.1 (7) °, respectively.

In the crystal packing, the molecules pack into layers parallel to (0 1 0) with connections between the layers provided by C–H···π, Table 1, and π–π interactions between the five-membered tetrazolo and pyrrole rings [Cg(N1–N4,C2)···Cg(N6,C3–C6)^i^ = 3.608 (3) Å, angle between planes = 5.0 (3) ° for *i*: 1-*x*, -*y*, -1/2+*z*], Fig. 2.

The Semi-empirical Quantum Chemical Calculations were performed on the experimental structure using the MOPAC2009 programme (Stewart, 2009) to optimize the structure with the Parametrization Model 6 (PM6) approximation together with the restricted Hartree-Fock closed-shell wavefunction; minimizations were terminated at an r.m.s. gradient of less than 0.01 kJ mol^-1^ Å^-1^. The most significant difference between the experimental and calculated structures is found in the relative orientation of the 4-methoxyphenyl ring with respect to the pyrrol ring to which it is bonded. This is quantified in the C3–C6–C7–C8 torsion angle of 38.9 ° *cf*. 24.2 (9) ° in the experimental structure. The orientation of the pyrrole-benzene ring remains unaffected.as seen in the (torsion angles is C4–N6–C14–C15 torsion angle of -38.8 ° *cf*. -39.1 (7) ° (experiment).

### Experimental

To a well stirred mixture of 5-(4-methoxyphenyl)-7-phenyl-4-chloro-7*H*-pyrrolo[2,3-d]pyrimidine (5 mmol) and Aliquat 336 (0.5 mmol) in toluene (25 ml) was added sodium azide (6 mmol) in water (5 ml). The reaction mixture was stirred under reflux conditions for 1.5 h. Thereafter, the two phases were separated. The aqueous phase was extracted with toluene and the combined organic layers were washed with water. The excess solvent was distilled off under reduced pressure. The obtained solid was dried to yield (I) which was crystallized from dioxane to obtain the final product (70 % yield, m.pt. 489–491 K). The crystals used for X-ray crystallography were obtained by slow evaporation from the an ethanol solution of (I).

### Refinement

The C-bound H atoms were geometrically placed (C–H = 0.93–0.96 Å) and refined as riding with *U_iso_*(H) = 1.2–1.5*U_eq_*(parent atom). In the absence of significant anomalous scattering effects, 1378 Friedel pairs were averaged in the final refinement.

### Crystal data

**Table:**

C_19_H_14_N_6_O	*F*(000) = 712
*M**_r_* = 342.36	*D*_x_ = 1.440 Mg m^−^^3^
Orthorhombic, *P**n**a*2_1_	Mo *K*α radiation, λ = 0.71073 Å
Hall symbol: P 2c -2n	Cell parameters from 2330 reflections
*a* = 9.3537 (7) Å	θ = 2.0–32.0°
*b* = 23.6045 (19) Å	µ = 0.10 mm^−^^1^
*c* = 7.1543 (6) Å	*T* = 293 K
*V* = 1579.6 (2) Å^3^	Block, colourless
*Z* = 4	0.35 × 0.25 × 0.20 mm

### Data collection

**Table:**

Bruker Kappa APEXII CCD diffractometer	1666 independent reflections
Radiation source: fine-focus sealed tube	1344 reflections with *I* > 2σ(*I*)
graphite	*R*_int_ = 0.051
ω and φ scan	θ_max_ = 25.9°, θ_min_ = 1.7°
Absorption correction: multi-scan (*SADABS*; Sheldrick, 1996)	*h* = −11→10
*T*_min_ = 0.967, *T*_max_ = 0.981	*k* = −28→26
16155 measured reflections	*l* = −8→8

### Refinement

**Table:**

Refinement on *F*^2^	Primary atom site location: structure-invariant direct methods
Least-squares matrix: full	Secondary atom site location: difference Fourier map
*R*[*F*^2^ > 2σ(*F*^2^)] = 0.041	Hydrogen site location: inferred from neighbouring sites
*wR*(*F*^2^) = 0.142	H-atom parameters constrained
*S* = 1.12	*w* = 1/[σ^2^(*F*_o_^2^) + (0.0924*P*)^2^ + 0.1003*P*] where *P* = (*F*_o_^2^ + 2*F*_c_^2^)/3
1666 reflections	(Δ/σ)_max_ = 0.001
236 parameters	Δρ_max_ = 0.50 e Å^−^^3^
1 restraint	Δρ_min_ = −0.27 e Å^−^^3^

### Special details

**Table:**

Geometry. All s.u.'s (except the s.u. in the dihedral angle between two l.s. planes) are estimated using the full covariance matrix. The cell s.u.'s are taken into account individually in the estimation of s.u.'s in distances, angles and torsion angles; correlations between s.u.'s in cell parameters are only used when they are defined by crystal symmetry. An approximate (isotropic) treatment of cell s.u.'s is used for estimating s.u.'s involving l.s. planes.
Refinement. Refinement of *F*^2^ against ALL reflections. The weighted *R*-factor wR and goodness of fit *S* are based on *F*^2^, conventional *R*-factors *R* are based on *F*, with *F* set to zero for negative *F*^2^. The threshold expression of *F*^2^ > 2σ(*F*^2^) is used only for calculating *R*-factors(gt) etc. and is not relevant to the choice of reflections for refinement. *R*-factors based on *F*^2^ are statistically about twice as large as those based on *F*, and *R*- factors based on ALL data will be even larger.

### Fractional atomic coordinates and isotropic or equivalent isotropic displacement parameters (Å^2^)

**Table:**

	*x*	*y*	*z*	*U*_iso_*/*U*_eq_	
O1	0.1311 (2)	−0.24532 (10)	0.1991 (5)	0.0436 (7)	
N1	0.6887 (3)	0.03688 (12)	0.2217 (6)	0.0388 (7)	
N2	0.8226 (3)	0.01376 (15)	0.2170 (8)	0.0538 (9)	
N3	0.8028 (3)	−0.04038 (15)	0.2108 (8)	0.0570 (10)	
N4	0.6627 (3)	−0.05488 (13)	0.2095 (7)	0.0490 (9)	
N5	0.5225 (3)	0.11037 (11)	0.2330 (6)	0.0394 (8)	
N6	0.2784 (3)	0.07783 (11)	0.2260 (5)	0.0345 (7)	
C1	0.6538 (4)	0.09319 (16)	0.2303 (7)	0.0434 (9)	
H1	0.7269	0.1199	0.2342	0.052*	
C2	0.5911 (3)	−0.00622 (14)	0.2167 (8)	0.0368 (8)	
C3	0.4447 (3)	0.00972 (13)	0.2204 (7)	0.0326 (7)	
C4	0.4226 (3)	0.06828 (13)	0.2276 (7)	0.0338 (8)	
C5	0.2112 (3)	0.02605 (13)	0.2160 (7)	0.0374 (8)	
H5	0.1126	0.0211	0.2128	0.045*	
C6	0.3086 (3)	−0.01734 (14)	0.2112 (7)	0.0348 (8)	
C7	0.2701 (3)	−0.07795 (14)	0.2027 (7)	0.0336 (8)	
C8	0.3593 (4)	−0.12011 (15)	0.2688 (6)	0.0413 (11)	
H8	0.4494	−0.1105	0.3136	0.050*	
C9	0.3172 (4)	−0.17651 (15)	0.2697 (7)	0.0430 (11)	
H9	0.3781	−0.2043	0.3161	0.052*	
C10	0.1848 (4)	−0.19110 (13)	0.2015 (7)	0.0356 (8)	
C11	0.0954 (4)	−0.14989 (15)	0.1288 (6)	0.0380 (9)	
H11	0.0071	−0.1597	0.0786	0.046*	
C12	0.1390 (4)	−0.09411 (16)	0.1317 (7)	0.0382 (9)	
H12	0.0782	−0.0665	0.0842	0.046*	
C13	0.2110 (4)	−0.28780 (15)	0.2954 (7)	0.0487 (11)	
H13A	0.3060	−0.2896	0.2449	0.073*	
H13B	0.1650	−0.3239	0.2802	0.073*	
H13C	0.2157	−0.2785	0.4259	0.073*	
C14	0.2042 (3)	0.13107 (13)	0.2232 (7)	0.0339 (8)	
C15	0.2549 (4)	0.17606 (15)	0.3270 (7)	0.0375 (9)	
H15	0.3365	0.1721	0.4002	0.045*	
C16	0.1822 (4)	0.22714 (15)	0.3202 (7)	0.0448 (10)	
H16	0.2173	0.2582	0.3860	0.054*	
C17	0.0586 (4)	0.23249 (15)	0.2172 (8)	0.0462 (9)	
H17	0.0097	0.2668	0.2137	0.055*	
C18	0.0080 (4)	0.18610 (15)	0.1186 (7)	0.0456 (10)	
H18	−0.0765	0.1892	0.0511	0.055*	
C19	0.0800 (4)	0.13608 (15)	0.1191 (7)	0.0405 (9)	
H19	0.0461	0.1055	0.0502	0.049*	

### Atomic displacement parameters (Å^2^)

**Table:**

	*U*^11^	*U*^22^	*U*^33^	*U*^12^	*U*^13^	*U*^23^
O1	0.0420 (14)	0.0314 (12)	0.057 (2)	−0.0069 (10)	−0.0019 (15)	0.0016 (15)
N1	0.0246 (14)	0.0442 (16)	0.048 (2)	0.0005 (12)	−0.0045 (17)	0.0019 (19)
N2	0.0259 (15)	0.062 (2)	0.073 (3)	0.0029 (14)	0.003 (2)	0.000 (3)
N3	0.0294 (16)	0.057 (2)	0.085 (3)	0.0061 (14)	0.005 (2)	0.000 (3)
N4	0.0281 (15)	0.0476 (19)	0.071 (3)	0.0056 (12)	0.0001 (18)	−0.002 (2)
N5	0.0326 (15)	0.0351 (15)	0.050 (2)	−0.0048 (12)	−0.0031 (17)	0.0032 (18)
N6	0.0292 (14)	0.0285 (14)	0.046 (2)	0.0018 (10)	−0.0029 (17)	0.0011 (16)
C1	0.0353 (19)	0.044 (2)	0.051 (3)	−0.0068 (15)	−0.003 (2)	0.002 (2)
C2	0.0297 (16)	0.0390 (18)	0.042 (2)	0.0006 (14)	0.001 (2)	−0.001 (2)
C3	0.0287 (16)	0.0324 (16)	0.0366 (19)	0.0006 (13)	0.001 (2)	−0.001 (2)
C4	0.0301 (16)	0.0333 (17)	0.038 (2)	−0.0008 (13)	−0.0004 (18)	0.0011 (19)
C5	0.0277 (16)	0.0322 (18)	0.052 (2)	−0.0003 (13)	0.000 (2)	−0.002 (2)
C6	0.0284 (16)	0.0319 (17)	0.044 (2)	0.0006 (13)	0.000 (2)	0.004 (2)
C7	0.0290 (17)	0.0308 (17)	0.041 (2)	0.0017 (13)	0.0057 (18)	−0.0007 (18)
C8	0.0305 (19)	0.038 (2)	0.055 (3)	−0.0003 (15)	−0.0042 (17)	−0.0009 (18)
C9	0.034 (2)	0.0303 (18)	0.065 (3)	0.0055 (15)	−0.0018 (19)	0.0029 (18)
C10	0.0358 (18)	0.0299 (17)	0.041 (2)	−0.0039 (13)	0.0079 (19)	−0.0023 (18)
C11	0.0292 (17)	0.042 (2)	0.042 (2)	−0.0046 (15)	−0.0006 (17)	0.0028 (19)
C12	0.0294 (18)	0.0359 (19)	0.049 (2)	0.0050 (15)	0.0026 (18)	0.0034 (18)
C13	0.059 (3)	0.0315 (19)	0.055 (3)	0.0006 (18)	0.003 (2)	0.0022 (19)
C14	0.0313 (17)	0.0298 (16)	0.041 (2)	0.0008 (13)	0.002 (2)	0.0026 (19)
C15	0.0334 (19)	0.0349 (19)	0.044 (2)	0.0012 (15)	−0.0025 (17)	0.0013 (18)
C16	0.048 (2)	0.035 (2)	0.051 (3)	0.0011 (17)	0.002 (2)	−0.0088 (19)
C17	0.047 (2)	0.0377 (19)	0.054 (3)	0.0110 (15)	0.003 (2)	0.001 (2)
C18	0.038 (2)	0.043 (2)	0.056 (3)	0.0061 (16)	−0.010 (2)	0.004 (2)
C19	0.038 (2)	0.0316 (18)	0.052 (3)	−0.0037 (15)	−0.006 (2)	−0.0005 (18)

### Geometric parameters (Å, °)

**Table:**

O1—C10	1.375 (4)	C8—C9	1.388 (5)
O1—C13	1.428 (5)	C8—H8	0.9300
N1—N2	1.367 (4)	C9—C10	1.374 (5)
N1—C2	1.367 (4)	C9—H9	0.9300
N1—C1	1.370 (5)	C10—C11	1.384 (5)
N2—N3	1.292 (5)	C11—C12	1.379 (5)
N3—N4	1.355 (4)	C11—H11	0.9300
N4—C2	1.330 (4)	C12—H12	0.9300
N5—C1	1.294 (4)	C13—H13A	0.9600
N5—C4	1.364 (4)	C13—H13B	0.9600
N6—C4	1.368 (4)	C13—H13C	0.9600
N6—C5	1.376 (4)	C14—C15	1.380 (5)
N6—C14	1.435 (4)	C14—C19	1.385 (5)
C1—H1	0.9300	C15—C16	1.385 (5)
C2—C3	1.420 (4)	C15—H15	0.9300
C3—C4	1.399 (5)	C16—C17	1.377 (6)
C3—C6	1.426 (4)	C16—H16	0.9300
C5—C6	1.371 (4)	C17—C18	1.386 (6)
C5—H5	0.9300	C17—H17	0.9300
C6—C7	1.477 (4)	C18—C19	1.359 (5)
C7—C12	1.380 (5)	C18—H18	0.9300
C7—C8	1.382 (5)	C19—H19	0.9300
			
C10—O1—C13	117.2 (3)	C10—C9—H9	120.2
N2—N1—C2	108.3 (3)	C8—C9—H9	120.2
N2—N1—C1	127.3 (3)	C9—C10—O1	124.5 (3)
C2—N1—C1	124.4 (3)	C9—C10—C11	120.1 (3)
N3—N2—N1	105.4 (3)	O1—C10—C11	115.4 (3)
N2—N3—N4	112.9 (3)	C12—C11—C10	119.1 (3)
C2—N4—N3	105.6 (3)	C12—C11—H11	120.4
C1—N5—C4	114.9 (3)	C10—C11—H11	120.4
C4—N6—C5	107.7 (3)	C11—C12—C7	122.1 (3)
C4—N6—C14	128.4 (3)	C11—C12—H12	118.9
C5—N6—C14	123.8 (3)	C7—C12—H12	118.9
N5—C1—N1	122.0 (3)	O1—C13—H13A	109.5
N5—C1—H1	119.0	O1—C13—H13B	109.5
N1—C1—H1	119.0	H13A—C13—H13B	109.5
N4—C2—N1	107.9 (3)	O1—C13—H13C	109.5
N4—C2—C3	135.6 (3)	H13A—C13—H13C	109.5
N1—C2—C3	116.5 (3)	H13B—C13—H13C	109.5
C4—C3—C2	113.9 (3)	C15—C14—C19	120.8 (3)
C4—C3—C6	108.2 (3)	C15—C14—N6	120.0 (3)
C2—C3—C6	137.8 (3)	C19—C14—N6	119.2 (3)
N5—C4—N6	123.7 (3)	C14—C15—C16	118.8 (4)
N5—C4—C3	128.3 (3)	C14—C15—H15	120.6
N6—C4—C3	107.9 (3)	C16—C15—H15	120.6
C6—C5—N6	111.2 (3)	C17—C16—C15	120.7 (4)
C6—C5—H5	124.4	C17—C16—H16	119.6
N6—C5—H5	124.4	C15—C16—H16	119.6
C5—C6—C3	104.9 (3)	C16—C17—C18	119.1 (3)
C5—C6—C7	124.2 (3)	C16—C17—H17	120.4
C3—C6—C7	130.9 (3)	C18—C17—H17	120.4
C12—C7—C8	117.6 (3)	C19—C18—C17	121.1 (4)
C12—C7—C6	120.0 (3)	C19—C18—H18	119.5
C8—C7—C6	122.4 (3)	C17—C18—H18	119.5
C7—C8—C9	121.4 (3)	C18—C19—C14	119.4 (4)
C7—C8—H8	119.3	C18—C19—H19	120.3
C9—C8—H8	119.3	C14—C19—H19	120.3
C10—C9—C8	119.6 (3)		
			
C2—N1—N2—N3	−0.4 (7)	C2—C3—C6—C5	−177.7 (6)
C1—N1—N2—N3	179.5 (5)	C4—C3—C6—C7	−179.5 (5)
N1—N2—N3—N4	0.5 (8)	C2—C3—C6—C7	3.6 (11)
N2—N3—N4—C2	−0.5 (7)	C5—C6—C7—C12	23.8 (8)
C4—N5—C1—N1	0.3 (7)	C3—C6—C7—C12	−157.7 (5)
N2—N1—C1—N5	179.6 (5)	C5—C6—C7—C8	−154.3 (5)
C2—N1—C1—N5	−0.5 (8)	C3—C6—C7—C8	24.2 (9)
N3—N4—C2—N1	0.2 (6)	C12—C7—C8—C9	−2.1 (7)
N3—N4—C2—C3	−179.7 (7)	C6—C7—C8—C9	176.0 (4)
N2—N1—C2—N4	0.1 (6)	C7—C8—C9—C10	0.8 (7)
C1—N1—C2—N4	−179.8 (5)	C8—C9—C10—O1	−178.6 (4)
N2—N1—C2—C3	−179.9 (5)	C8—C9—C10—C11	1.3 (7)
C1—N1—C2—C3	0.2 (8)	C13—O1—C10—C9	8.3 (6)
N4—C2—C3—C4	−179.7 (6)	C13—O1—C10—C11	−171.7 (4)
N1—C2—C3—C4	0.3 (7)	C9—C10—C11—C12	−2.1 (7)
N4—C2—C3—C6	−2.9 (12)	O1—C10—C11—C12	177.8 (4)
N1—C2—C3—C6	177.1 (6)	C10—C11—C12—C7	0.8 (7)
C1—N5—C4—N6	−178.8 (5)	C8—C7—C12—C11	1.3 (7)
C1—N5—C4—C3	0.2 (8)	C6—C7—C12—C11	−176.9 (4)
C5—N6—C4—N5	178.6 (5)	C4—N6—C14—C15	−39.1 (7)
C14—N6—C4—N5	2.3 (8)	C5—N6—C14—C15	145.1 (4)
C5—N6—C4—C3	−0.6 (5)	C4—N6—C14—C19	142.5 (5)
C14—N6—C4—C3	−176.9 (5)	C5—N6—C14—C19	−33.3 (7)
C2—C3—C4—N5	−0.6 (8)	C19—C14—C15—C16	−2.3 (6)
C6—C3—C4—N5	−178.3 (5)	N6—C14—C15—C16	179.3 (4)
C2—C3—C4—N6	178.6 (4)	C14—C15—C16—C17	2.3 (7)
C6—C3—C4—N6	0.9 (6)	C15—C16—C17—C18	−0.5 (7)
C4—N6—C5—C6	0.1 (5)	C16—C17—C18—C19	−1.4 (7)
C14—N6—C5—C6	176.6 (5)	C17—C18—C19—C14	1.4 (7)
N6—C5—C6—C3	0.4 (6)	C15—C14—C19—C18	0.5 (7)
N6—C5—C6—C7	179.2 (5)	N6—C14—C19—C18	178.8 (4)
C4—C3—C6—C5	−0.8 (6)		

### Hydrogen-bond geometry (Å, °)

**Table:**

Cg1 is the centroid of the C14–C19 ring.

**Table:**

*D*—H···*A*	*D*—H	H···*A*	*D*···*A*	*D*—H···*A*
C8—H8···N4	0.93	2.50	3.257 (5)	138
C15—H15···N5	0.93	2.57	3.020 (5)	111
C11—H11···Cg1^i^	0.93	2.91	3.684 (5)	141
C13—H13c···Cg1^ii^	0.96	2.72	3.459 (5)	134

Symmetry codes:  (i) −*x*, −*y*, *z*−1/2; (ii) −*x*+1/2, *y*−1/2, *z*+1/2.
